# Prevalence of co-existing autoimmune disease in juvenile idiopathic arthritis: a cross-sectional study

**DOI:** 10.1186/s12969-020-00426-9

**Published:** 2020-06-05

**Authors:** Teresa A. Simon, Gowri Priya Harikrishnan, Hugh Kawabata, Sanket Singhal, Hermine I. Brunner, Daniel J. Lovell

**Affiliations:** 1 Bristol Myers Squibb, Princeton, NJ USA; 2 Mu Sigma, Bangalore, India; 3 Cincinnati Children’s Hospital Medical Center, Cincinnati, OH USA

**Keywords:** ADHD, Comorbidity, Co-existing autoimmune disease, Epidemiology, Juvenile idiopathic arthritis, Prevalence

## Abstract

**Background:**

Many autoimmune diseases share common pathogenic mechanisms, cytokine pathways and systemic inflammatory cascades; however, large studies quantifying the co-existence of autoimmune diseases in patients with juvenile idiopathic arthritis (JIA) have not been conducted.

**Methods:**

We performed a cross-sectional study using two United States administrative healthcare claims databases (Truven Health MarketScan® Commercial Database and IMS PharMetrics database) to screen for the prevalence of multiple autoimmune diseases in patients with JIA and in a control group with attention deficit hyperactivity disorder (ADHD). Patients with a diagnosis code for JIA or ADHD between January 1, 2006 and September 30, 2017 were separated into two age cohorts (< 18 and ≥ 18 years) and matched (maximum 1:5) based on age, sex, number of medical encounters, and calendar year of diagnosis. The prevalence rates of 30 pre-specified autoimmune diseases during the 12-month periods before and after diagnosis were compared.

**Results:**

Overall, 29,215 patients with JIA and 134,625 matched control patients with ADHD were evaluated. Among patients in the MarketScan database, 28/30 autoimmune diseases were more prevalent in patients with JIA aged < 18 years and 29/30 were more prevalent in patients aged ≥ 18 years when compared with a matched cohort of patients with ADHD. In the PharMetrics database, 29/30 and 30/30 autoimmune diseases were more prevalent in patients with JIA aged < 18 and ≥ 18 years, respectively, compared with a matched cohort of patients with ADHD. Among patients with JIA aged < 18 years, the greatest odds ratios (ORs) were seen for Sjögren’s syndrome/sicca syndrome and uveitis. Among patients aged ≥ 18 years in the MarketScan database, the greatest ORs were recorded for uveitis. Data from the PharMetrics database indicated that the greatest ORs were for uveitis and chronic glomerulonephritis.

**Conclusions:**

Patients with JIA are more likely to have concurrent autoimmune diseases than matched patients with ADHD. Having an awareness of the co-existence of autoimmune diseases among patients with JIA may play an important role in patient management, treatment decisions, and outcomes.

**Trial registration:**

Not applicable.

## Background

Autoimmune diseases are the most prevalent disease conditions reported in the United States (US) [1]. The overall estimated prevalence rate of autoimmune disease is 3.1% in the US [2] and 7.6–9.4% in Europe [3], depending on the source and if correction for under-ascertainment is used. Research has demonstrated that the incidence and prevalence of autoimmune diseases have been steadily and significantly rising over the past three decades [4]. This is of particular concern as autoimmune diseases have been associated with increases in morbidity and mortality [5, 6].

There are over 100 known autoimmune diseases [7] and many exhibit shared pathogenic mechanisms [8], reflecting a complex interaction between environmental and genetic factors [9]. Genome-wide association studies have increased the understanding of the shared cytokine pathways and systemic inflammatory cascades characteristic of autoimmune diseases [8, 9]. Despite being in an era of electronic medical records and integrated care, the US remains mostly a siloed healthcare system [10]. Therefore, as autoimmune diseases span a number of medical specialties, it is important to raise awareness of those that overlap disciplines in order to improve patient care.

Juvenile idiopathic arthritis (JIA) is the most common chronic pediatric rheumatic disease and is characterized by joint inflammation that often leads to joint damage, chronic pain and disability [11, 12]. The term ‘JIA’ encompasses seven clinically heterogeneous groups of arthritides of unknown cause in children [11]. JIA is not confined to childhood, with more than a third of patients continuing to have episodes of active inflammation during their adult years [13, 14]. Several studies have reported the prevalence of JIA; however, there is limited knowledge about the frequency of the co-occurrence of other autoimmune diseases in patients with JIA [15, 16], with the exception of uveitis, which is known to occur in 12–31% of patients with JIA [17, 18]. In addition, for JIA, the majority of publications are case reports [19–21] or small clinical/hospital studies [22–28] involving one or a select few autoimmune diseases. These studies show that co-existing autoimmune diseases, particularly thyroid conditions, are common in patients with JIA. Previous studies have evaluated the co-existence of other autoimmune diseases in patients with psoriasis [29] and adult patients with rheumatoid arthritis (RA) [15] and multiple sclerosis [30].

Comorbid conditions in patients with JIA, including co-existing autoimmune diseases, negatively impact quality of life, disability, and mortality [31, 32]. In a single-center study of 79 patients with JIA, 10.1% had one additional autoimmune disease and 5.1% had two [28]. As such, understanding the presence of overlapping autoimmune diseases is important in developing treatment strategies, particularly as some autoimmune diseases can arise or be worsened by common treatments, such as tumor necrosis factor inhibitors, which are commonly used in the treatment of diseases such as JIA and RA [8, 33, 34].

The aim of this large, cross-sectional study was to quantify the co-occurrence of autoimmune diseases in patients with JIA. In addition, this study evaluated the distribution of these autoimmune diseases based on age (< 18 and ≥ 18 years). In a database study, among patients with JIA who transferred from pediatric to adult rheumatology care after the age of 14 years, the median age at transfer was 18 years [35]. To address potential observation bias, the prevalence rates of co-existing autoimmune diseases were also evaluated in patients with attention deficit hyperactivity disorder (ADHD, a common disorder generally diagnosed in childhood that often persists into adulthood [36]), who have similarly frequent interactions with the healthcare system (e.g. healthcare encounter) as do patients with JIA [37].

## Methods

### Study design

This was a large, retrospective, cross-sectional study using two US administrative healthcare claims databases: the Truven Health MarketScan® Commercial Database (MarketScan®) and the IMS PharMetrics database (PharMetrics). MarketScan includes data from 2006 onward for over 70 million privately insured patients under the age of 65 years. PharMetrics contains claims information on approximately 55 million patients from over 90 managed care plans and other sources from 2007 onward. These databases contain insurance claim information that includes anonymized information on diagnoses associated with physician visits, medical procedures, hospitalizations, prescribed medications, and laboratory tests for insured individuals.

A common data model was used to standardize the data structure and terminologies of the two databases in order to minimize the need for customizations and allow the proposed analytic methods to be applied systematically. The use of this model allows the source data to be represented more reliably [38].

The study was conducted in accordance with the International Society for Pharmacoepidemiology Guidelines for Good Pharmacoepidemiology Practices [39], applicable regulatory requirements and the principles of the Declaration of Helsinki. No new studies with human or animal subjects are reported in this article. Review and approval by ethics committees and patient informed consent were not required for this study because data were anonymized at the source.

### Patients

Patients with a diagnosis code for JIA or ADHD between January 1, 2006 and September 30, 2017 were included in this study; this was not necessarily the first time the patient was diagnosed with JIA or ADHD. Patients were required to have two occurrences of an International Classification of Diseases, Ninth Revision, Clinical Modification (ICD-9-CM) diagnosis code of 714.3x or International Classification of Diseases, Tenth Revision, Clinical Modification (ICD-10-CM) diagnosis code of M08.x for JIA and 314.0x (ICD-9-CM) and F90.x (ICD-10-CM) for ADHD on separate occasions within a 90-day period. At least 1 year of continuous health plan enrollment before the qualifying JIA or ADHD diagnosis code and 1 year after the diagnosis code were required for inclusion in this study. Patients were stratified into four cohorts: < 18-year-old patients with JIA, ≥ 18-year-old patients with JIA, < 18-year-old patients with ADHD, and ≥ 18-year-old patients with ADHD. Age was categorized based on age of the patient at the time the qualifying JIA/ADHD diagnosis code was reported. Patients with both JIA and ADHD or > 3 medical encounters listed in 1 day were excluded.

### Outcomes

Prevalence rates of 30 common autoimmune conditions (Supplementary Table 1) were investigated among patients with JIA or ADHD during the 1-year baseline (when the first qualifying JIA/ADHD diagnosis code was reported) and 1-year follow-up periods. RA was included as an outcome not to observe if it co-existed with JIA, but to ascertain whether a greater proportion of patients with JIA aged ≥ 18 years, compared with those < 18 years, also received a diagnosis of RA.

### Statistical analyses

Patients with JIA were frequency matched to patients with ADHD for each combination of the following characteristics: age (± 2 years), sex, number of medical encounters (in-patient, out-patient, and emergency room visits), and calendar year of diagnosis (± 1 year). For each patient with JIA, up to 5 matched patients with ADHD were identified. Matching was carried out with replacement for the ADHD group; patients with ADHD could be matched to a maximum of 7 (PharMetrics) or 8 (MarketScan) patients with JIA.

Descriptive statistics were used to analyze baseline demographic information, comorbid conditions, and medication use. The co-occurrence of autoimmune diseases was studied using a univariate analysis to understand their individual association with JIA/ADHD. Data are presented as matched prevalence rates, 95% confidence intervals and odds ratios (ORs) for patients who had two relevant ICD-9-CM or ICD-10-CM diagnosis codes for any of the 30 pre-specified autoimmune diseases within 1 year before and after the qualifying JIA/ADHD diagnosis code was recorded. Descriptive statistics (%) were used to analyze the reported prevalences of RA, ankylosing spondylitis, psoriatic arthritis, dermatomyositis, systemic lupus erythematosus (SLE), and sarcoidosis as a diagnosis code for these diseases does not represent co-existence, but more likely misclassification. Results presented by age group are shown for the patients’ ages at the time of the study.

## Results

### Patient disposition and baseline characteristics

**Fig. 1:**
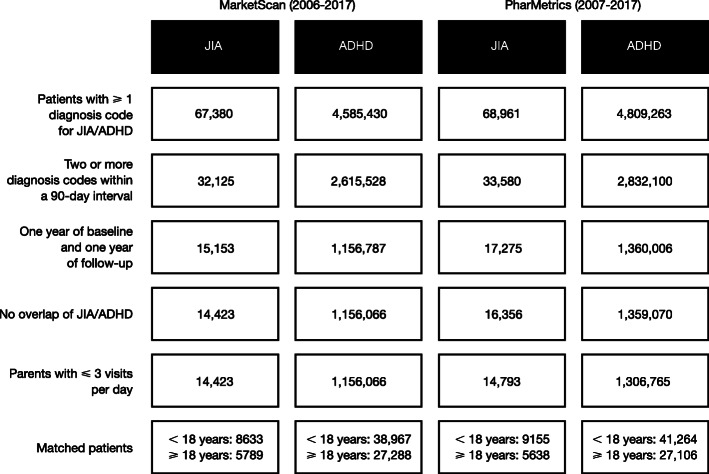
Patient disposition. Cells show number of patients. *ADHD* attention deficit hyperactivity disorder, *JIA* juvenile idiopathic arthritis*, MarketScan* Truven Health MarketScan® Commercial Database, *PharMetrics* IMS PharMetrics database

A total of 30,779 patients with JIA and 2,515,136 patients with ADHD were eligible for inclusion across the two databases (Fig. 1). After matching and removal of those with excess matches (> 5) or excess encounters (> 3), 29,215 patients with JIA and 134,625 patients with ADHD were analyzed. Across both databases and age cohorts, 100% of patients with JIA were included.

The baseline characteristics of each individual cohort were similar before (Supplementary Table 2) and after matching (Table 1), with the exception of sex and number of medical encounters. These differences were accounted for with the frequency matching analyses. In the matched patients, across all cohorts, approximately three-quarters of patients were female (range: 69–78%), with a mean age of 10.5–11 years in the < 18 years cohort and 34–37 years in the ≥ 18 years cohort at the time of the qualifying JIA/ADHD diagnosis code (Table 1). Across both age cohorts, a greater proportion of patients with JIA were receiving disease-modifying antirheumatic drugs (DMARDs), corticosteroids, or non-steroidal anti-inflammatory drugs compared with those with ADHD. Among patients with JIA, 23–25% of those aged < 18 years and 52–54% aged ≥ 18 years also had a diagnosis of RA. This is expected, as non-pediatric rheumatologist physicians may give a diagnosis of RA, in particular when a patient with JIA moves from pediatric care to an adult healthcare setting [41, 42]. Diagnoses of psoriatic arthritis and ankylosing spondylitis, respectively, were reported in 3 and 1% of patients with JIA aged < 18 years and 1–2 and 2% aged ≥ 18 years. Psoriatic arthritis and ankylosing spondylitis were not considered as co-existing autoimmune diseases because the diagnosis codes for these autoimmune diseases may be used for specific subcategories of JIA. Dermatomyositis, SLE, and sarcoidosis, respectively, were reported in 0.5%, 0.8–0.9%, and 0.1% of patients with JIA aged < 18 years and 0.3%, 2–3% and 0.4% aged ≥ 18 years. As these diseases present with symptoms that overlap with JIA, which may lead potential errors in diagnoses codes, these diseases were not considered co-existing.

**Table 1:** Baseline characteristics after matching for JIA and ADHD groups

Characteristic	< 18 years				≥ 18 years			
	MarketScan		PharMetrics		MarketScan		PharMetrics	
	JIA (*N* = 8633)	ADHD (*N* = 38,967)	JIA (*N* = 9155)	ADHD (*N* = 41,264)	JIA (*N* = 5789)	ADHD (*N* = 27,288)	JIA (*N* = 5638)	ADHD (*N* = 27,106)
**Female**	6173 (71.5)	26,933 (69.1)	6587 (71.9)	28,694 (69.5)	4506 (77.8)	21,096 (77.3)	4315 (76.5)	20,590 (76.0)
**Age, mean (SD)**	10.6 (4.5)	11.1 (3.9)	10.5 (4.5)	11.0 (3.9)	37.0 (16.5)	36.2 (15.4)	34.6 (14.7)	34.4 (14.3)
**Medications**								
bDMARD	1287 (14.9)	9 (< 0.1)	1485 (16.2)	9 (< 0.1)	1593 (27.5)	121 (0.4)	1614 (28.6)	129 (0.5)
Non-bDMARD	2512 (29.1)	529 (1.4)	3127 (34.2)	534 (1.3)	2143 (37.0)	870 (3.2)	2266 (40.2)	845 (3.1)
Corticosteroids^a^	2984 (34.6)	7500 (19.2)	3367 (36.8)	4696 (11.4)	2681 (46.3)	7845 (28.7)	2712 (48.1)	8496 (31.3)
NSAIDs	4090 (47.4)	3037 (7.8)	4812 (52.6)	9868 (23.9)	2528 (43.7)	7391 (27.1)	2634 (46.7)	8101 (29.9)
**Comorbidities**								
Multiple sclerosis	1 (< 0.1)	11 (< 0.1)	1 (< 0.1)	8 (< 0.1)	20 (0.3)	87 (0.3)	19 (0.3)	65 (0.2)
Asthma	777 (9.0)	4130 (10.6)	887 (9.7)	5410 (13.1)	472 (8.2)	2279 (8.4)	486 (8.6)	2565 (9.5)
Anxiety	295 (3.4)	6123 (15.7)	317 (3.5)	7665 (18.6)	555 (9.6)	8098 (29.7)	549 (9.7)	9185 (33.9)
Depression	208 (2.4)	4478 (11.5)	214 (2.3)	5293 (12.8)	653 (11.3)	9469 (34.7)	622 (11.0)	9868 (36.4)
Diabetes mellitus	92 (1.1)	240 (0.6)	84 (0.9)	259 (0.6)	440 (7.6)	1408 (5.2)	371 (6.6)	1146 (4.2)
Anemia	498 (5.8)	540 (1.4)	479 (5.2)	741 (1.8)	948 (16.4)	1762 (6.5)	655 (11.6)	1674 (6.2)
Hypertension	2 (< 0.1)	11 (< 0.1)	6 (0.1)	9 (< 0.1)	103 (1.8)	268 (1.0)	103 (1.8)	319 (1.2)
Hyperlipidemia	174 (2.0)	987 (2.5)	202 (2.2)	1388 (3.4)	1162 (20.1)	5355 (19.6)	1023 (18.1)	5264 (19.4)
Malignancy	56 (0.6)	128 (0.3)	49 (0.5)	136 (0.3)	278 (4.8)	856 (3.1)	162 (2.9)	702 (2.6)
**CCI score, mean (SD)** [40]	2.4 (0.6)	2.1 (0.4)	2.4 (0.6)	2.1 (0.5)	2.9 (1.0)	2.3 (0.7)	2.8 (0.9)	2.2 (0.7)
**Autoimmune diseases**^b^								
0	4988 (57.8)	37,675 (96.7)	5194 (56.7)	39,680 (96.2)	1973 (34.1)	24,792 (90.9)	2029 (36.0)	24,739 (91.3)
1	2830 (32.8)	1220 (3.1)	3075 (33.6)	1510 (3.7)	2749 (47.5)	2160 (7.9)	2641 (46.8)	2052 (7.6)
2	659 (7.6)	63 (0.2)	745 (8.1)	67 (0.2)	796 (13.8)	283 (1.0)	722 (12.8)	261 (1.0)
3	127 (1.5)	8 (< 0.1)	121 (1.3)	7 (< 0.1)	190 (3.3)	42 (0.2)	190 (3.4)	42 (0.2)
> 3	29 (0.3)	1 (< 0.1)	20 (0.2)	0 (0.0)	81 (1.4)	11 (< 0.1)	56 (1.0)	12 (< 0.1)
**Healthcare encounters**^**c**^								
0–10	4927 (57.1)	22,969 (58.9)	3205 (35.0)	15,400 (37.3)	2713 (46.9)	13,157 (48.2)	1656 (29.4)	8139 (30.0)
11–20	2353 (27.3)	9995 (25.6)	3352 (36.6)	14,630 (35.5)	1567 (27.1)	7352 (26.9)	1741 (30.9)	8371 (30.9)
21–30	739 (8.6)	3235 (8.3)	1393 (15.2)	5930 (14.4)	730 (12.6)	3346 (12.3)	1008 (17.9)	4829 (17.8)
31–40	294 (3.4)	1318 (3.4)	604 (6.6)	2607 (6.3)	371 (6.4)	1677 (6.1)	561 (10.0)	2655 (9.8)
41–50	153 (1.8)	701 (1.8)	262 (2.9)	1150 (2.8)	183 (3.2)	821 (3.0)	273 (4.8)	1295 (4.8)
> 50	167 (1.9)	749 (1.9)	339 (3.7)	1547 (3.7)	225 (3.9)	935 (3.4)	399 (7.1)	1817 (6.7)

Data are shown as n (%) unless otherwise specified

^a^By any route of administration

^b^Includes all autoimmune diseases outlined in Supplementary Table 1, rheumatoid arthritis, ankylosing spondylitis, psoriatic arthritis, dermatomyositis, systemic lupus erythematosus, and sarcoidosis

^**c**^In 1 year

*ADHD* attention deficit hyperactivity disorder, *bDMARD* biologic disease-modifying antirheumatic drug, *CCI* Charlson Comorbidity Index [40], *JIA* juvenile idiopathic arthritis, *MarketScan* Truven Health MarketScan® Commercial Database, *NSAID* nonsteroidal anti-inflammatory drug, *PharMetrics* IMS PharMetrics database*, SD* standard deviation

All patient characteristics were similar between both healthcare claims databases, except number of medical encounters, which were higher in the PharMetrics cohort.

Differences in baseline comorbid conditions were noted between patients of all ages with JIA and ADHD (Table 1): patients with JIA were more likely to have anemia, whereas patients with ADHD were more likely to have anxiety or depression. In both the JIA and ADHD cohorts, patients in the < 18 years cohorts showed a lower rate of all comorbid conditions, except asthma, and also lower rates of DMARD and corticosteroid use compared with the ≥ 18 years cohort. The total number of co-existing autoimmune diseases identified among the patients with JIA was greater compared with those with ADHD for patients of all ages. Both patients with JIA and patients with ADHD aged ≥ 18 years were more likely to have more than one autoimmune disease than patients < 18 years with the same conditions.

### Prevalence

**Fig. 2:**
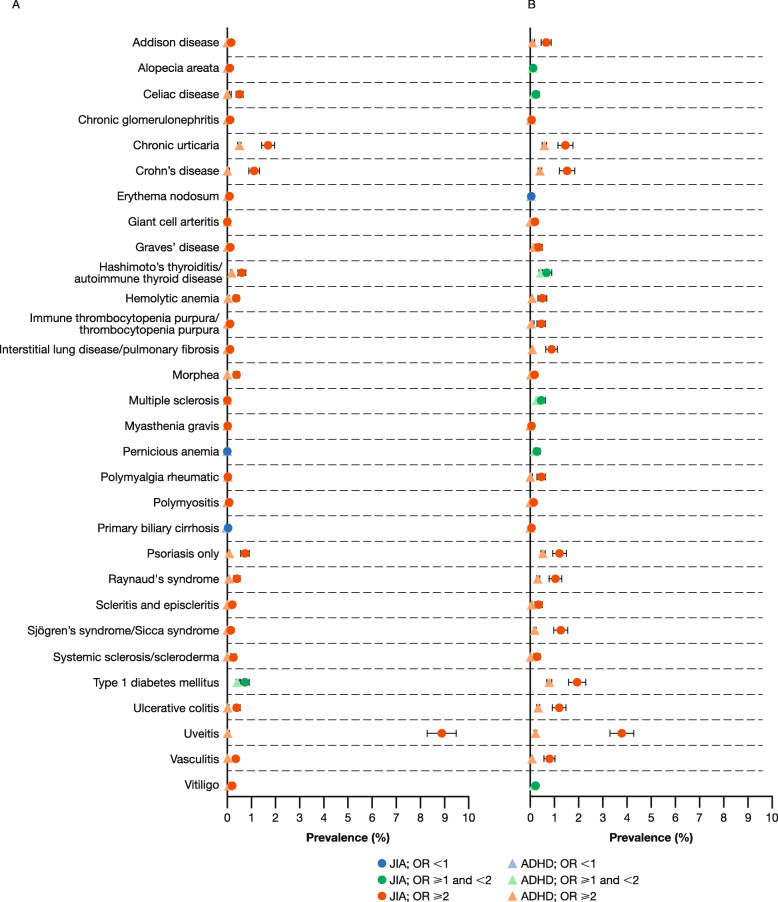
Autoimmune disease prevalence* among patients aged (**a**) < 18 and (**b**) ≥ 18 years: MarketScan. *Using two diagnosis codes. *ADHD* attention deficit hyperactivity disorder, *JIA* juvenile idiopathic arthritis, *MarketScan* Truven Health MarketScan® Commercial Database*, OR* odds ratio

**Fig. 3:**
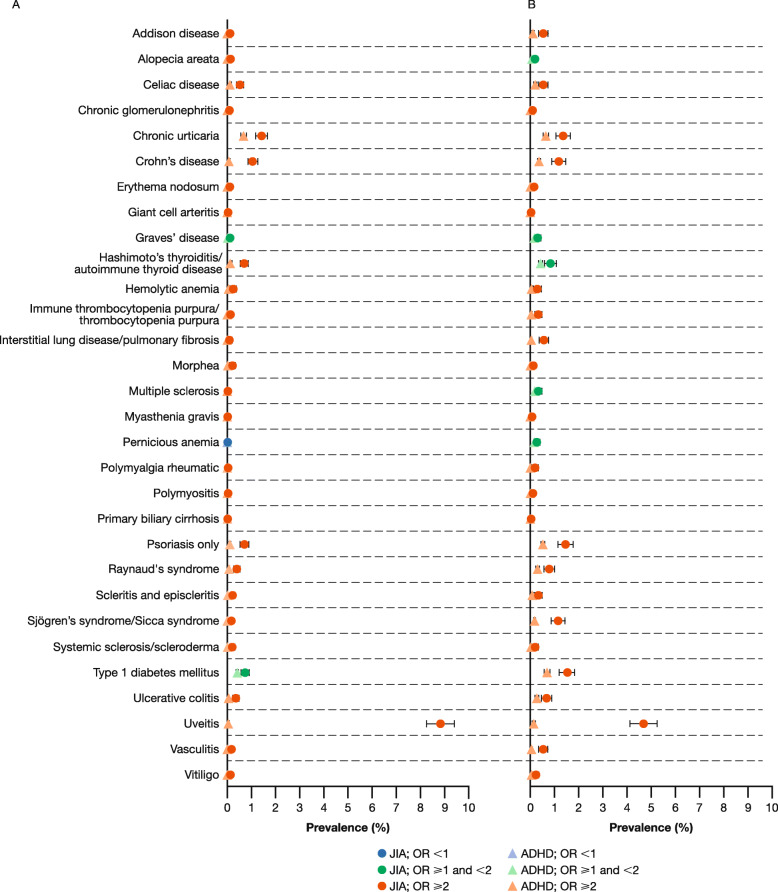
Autoimmune disease prevalence* among patients aged (**a**) < 18 and (**b**) ≥ 18 years: PharMetrics. *Using two diagnosis codes. *ADHD* attention deficit hyperactivity disorder, *JIA* juvenile idiopathic arthritis, *OR* odds ratio, *PharMetrics* IMS PharMetrics database

Overall, the prevalence rates of the pre-specified autoimmune diseases evaluated were higher among patients with JIA than among matched patients with ADHD (Figs. 2 and 3, Table 2), with few differences between the two databases. Among patients with JIA in MarketScan, 28/30 autoimmune diseases were more prevalent in patients with JIA aged < 18 years and 29/30 were more prevalent in patients aged ≥ 18 years, when compared with a matched cohort of patients with ADHD. In PharMetrics, 29/30 and 30/30 autoimmune diseases were more prevalent in patients with JIA aged < 18 and ≥ 18 years, respectively, compared with a matched cohort of patients with ADHD. For the patients with JIA, uveitis was the most prevalent co-existing autoimmune disease in both age groups. After uveitis, the most prevalent co-existing autoimmune disease among patients with JIA aged < 18 years was chronic urticaria. In patients with JIA aged ≥ 18 years, type 1 diabetes mellitus was the most prevalent. Conditions with similar rates among patients with JIA aged < 18 and ≥ 18 years included alopecia areata, chronic urticaria, Crohn’s disease, Hashimoto’s thyroiditis/autoimmune thyroid disease, and systemic sclerosis/scleroderma.

**Table 2:** Prevalence (%) and odds ratios for pre-specified autoimmune diseases, using two diagnosis codes

Prevalence (%)	< 18 years						≥ 18 years					
	MarketScan			PharMetrics			MarketScan			PharMetrics		
	JIA (*N* = 8633)	ADHD (*N* = 38,967)	Odds ratio (95% CI)	JIA (*N* = 9155)	ADHD (*N* = 41,264)	Odds ratio (95% CI)	JIA (*N* = 5789)	ADHD (*N* = 27,288)	Odds ratio (95% CI)	JIA (*N* = 5638)	ADHD (*N* = 27,106)	Odds ratio (95% CI)
Addison disease	0.17	0.03	6.16 (2.83, 13.42)	0.11	0.04	2.51 (1.16, 5.43)	0.66	0.14	4.87 (3.09, 7.66)	0.53	0.11	4.67 (2.83, 7.73)
Alopecia areata	0.10	0.05	2.03 (0.93, 4.46)	0.15	0.06	2.43 (1.27, 4.65)	0.10	0.10	1.09 (0.45, 2.64)	0.18	0.11	1.66 (0.81, 3.41)
Celiac disease	0.51	0.13	3.99 (2.66, 5.98)	0.54	0.14	3.82 (2.61, 5.60)	0.24	0.18	1.32 (0.73, 2.39)	0.53	0.20	2.63 (1.68, 4.11)
Chronic glomerulonephritis	0.12	0.00	Inf	0.08	0.00	31.57 (3.88, 256.56)	0.05	0.02	2.36 (0.59, 9.43)	0.09	0.00	24.06 (2.81, 205.96)
Chronic urticaria	1.69	0.50	3.40 (2.74, 4.22)	1.42	0.70	2.04 (1.65, 2.51)	1.45	0.58	2.53 (1.94, 3.30)	1.35	0.67	2.03 (1.55, 2.66)
Crohn’s disease	1.11	0.07	16.84 (10.91, 25.99)	1.06	0.08	12.99 (8.78, 19.2)	1.52	0.40	3.88 (2.93, 5.16)	1.17	0.37	3.23 (2.36, 4.42)
Erythema nodosum	0.09	0.01	12.05 (3.20, 45.4)	0.09	0.01	9.02 (2.72, 29.96)	0.03	0.04	0.94 (0.21, 4.3)	0.14	0.02	7.7 (2.52, 23.55)
Giant cell arteritis	0.01	0.00	4.51 (0.28, 72.18)	0.01	0.00	Inf	0.19	0.00	Inf	0.04	0.01	3.21 (0.54, 19.19)
Graves’ disease	0.12	0.04	3.23 (1.43, 7.27)	0.10	0.06	1.69 (0.79, 3.64)	0.35	0.16	2.15 (1.26, 3.65)	0.28	0.20	1.45 (0.83, 2.54)
Hashimoto’s thyroiditis/ autoimmune thyroid disease	0.60	0.17	3.57 (2.48, 5.14)	0.70	0.16	4.26 (3.03, 6.01)	0.67	0.43	1.56 (1.09, 2.25)	0.83	0.42	1.99 (1.42, 2.8)
Hemolytic anemia	0.37	0.05	6.90 (3.98, 11.97)	0.24	0.05	4.52 (2.50, 8.16)	0.50	0.07	6.86 (3.88, 12.14)	0.30	0.06	5.46 (2.73, 10.94)
Immune thrombocytopenia purpura/ thrombocytopenia purpura	0.12	0.03	4.52 (1.88, 10.86)	0.14	0.03	4.89 (2.23, 10.72)	0.45	0.11	3.97 (2.35, 6.69)	0.32	0.07	4.82 (2.51, 9.27)
Interstitial lung disease/pulmonary fibrosis	0.13	0.01	24.86 (5.51, 112.15)	0.09	0.01	12.03 (3.19, 45.33)	0.88	0.08	11.54 (6.94, 19.2)	0.57	0.04	12.89 (6.63, 25.04)
Morphea	0.38	0.02	21.36 (9.45, 48.29)	0.23	0.01	18.97 (7.15, 50.33)	0.17	0.05	3.63 (1.59, 8.28)	0.12	0.03	4.81 (1.69, 13.73)
Multiple sclerosis	0.01	0.01	2.26 (0.21, 24.90)	0.03	0.01	3.38 (0.76, 15.11)	0.45	0.30	1.5 (0.96, 2.33)	0.32	0.21	1.52 (0.89, 2.58)
Myasthenia gravis	0.02	0.01	4.51 (0.64, 32.06)	0.00	0.00	Inf	0.05	0.02	2.36 (0.59, 9.43)	0.07	0.02	3.85 (1.03, 14.34)
Pernicious anemia	0.00	0.01	0	0.00	0.00	0	0.28	0.21	1.3 (0.75, 2.27)	0.27	0.21	1.24 (0.7, 2.2)
Polymyalgia rheumatic	0.01	0.00	Inf	0.02	0.00	9.02 (0.82, 99.43)	0.45	0.05	8.2 (4.34, 15.5)	0.20	0.05	3.78 (1.72, 8.34)
Polymyositis	0.08	0.00	Inf	0.05	0.00	22.55 (2.63, 193.01)	0.14	0.02	7.55 (2.47, 23.09)	0.09	0.02	4.01 (1.22, 13.14)
Primary biliary cirrhosis	0.00	0.00	0	0.00	0.00	Inf	0.05	0.02	2.83 (0.68, 11.84)	0.02	0.01	2.40 (0.22, 26.52)
Psoriasis only	0.73	0.09	8.42 (5.54, 12.78)	0.71	0.12	5.89 (4.07, 8.53)	1.21	0.53	2.31 (1.73, 3.08)	1.45	0.50	2.93 (2.22, 3.86)
Raynaud’s syndrome	0.39	0.07	5.7 (3.44, 9.46)	0.64	0.05	12.16 (7.45, 19.84)	1.04	0.32	3.24 (2.33, 4.50)	0.78	0.31	2.53 (1.76, 3.65)
Scleritis and episcleritis	0.22	0.01	17.19 (6.42, 46.04)	0.23	0.02	13.55 (5.76, 31.88)	0.35	0.07	5.25 (2.78, 9.94)	0.34	0.09	3.66 (2.02, 6.66)
Sjögren’s syndrome /sicca syndrome	0.16	0.00	Inf	0.17	0.00	72.24 (9.58, 544.36)	1.26	0.19	6.82 (4.76, 9.77)	1.15	0.17	6.86 (4.70, 10.02)
Systemic sclerosis/scleroderma	0.25	0.01	24.89 (8.57, 72.23)	0.22	0.00	90.34 (12.12, 673.22)	0.28	0.03	10.8 (4.44, 26.26)	0.20	0.02	8.83 (3.26, 23.88)
Type 1 diabetes mellitus	0.73	0.43	1.7 (1.27, 2.27)	0.74	0.41	1.82 (1.37, 2.41)	1.93	0.78	2.50 (1.98, 3.14)	1.51	0.71	2.15 (1.66, 2.78)
Ulcerative colitis	0.41	0.05	8.34 (4.77, 14.59)	0.36	0.06	6.49 (3.81, 11.05)	1.19	0.33	3.69 (2.69, 5.06)	0.67	0.28	2.41 (1.63, 3.57)
Uveitis	8.88	0.06	165.10 (108.97, 250.14)	8.84	0.07	133.23 (92.47, 191.95)	3.78	0.21	18.46 (13.8, 24.69)	4.68	0.17	29.54 (21.51, 40.58)
Vasculitis	0.35	0.02	22.64 (9.42, 54.42)	0.17	0.02	10.32 (4.24, 25.08)	0.79	0.07	12.13 (7.03, 20.94)	0.53	0.07	7.63 (4.29, 13.56)
Vitiligo	0.14	0.05	2.85 (1.39, 5.88)	0.12	0.05	2.61 (1.24, 5.49)	0.16	0.08	1.85 (0.85, 3.99)	0.23	0.11	2.16 (1.12, 4.15)

*ADHD* attention deficit hyperactivity disorder, *CI* confidence interval, *Inf* infinite (due to the fact that no patients in the ADHD cohort reported any cases of the autoimmune disease), *JIA* juvenile idiopathic arthritis, *MarketScan* Truven Health MarketScan® Commercial Database, *PharMetrics* IMS PharMetrics database

Among patients with JIA aged < 18 years, across both databases the greatest ORs were seen for Sjögren’s syndrome/sicca syndrome and uveitis (Table 2). Among these patients, similar odds were observed for pernicious anemia and primary biliary cirrhosis when compared with the matched patients with ADHD. Among patients with JIA aged ≥ 18 years in MarketScan, the greatest ORs were recorded for uveitis; in PharMetrics, the greatest ORs were for uveitis and chronic glomerulonephritis.

Among patients of all ages with ADHD, the most prevalent autoimmune disease was chronic urticaria followed by type 1 diabetes. All autoimmune diseases studied were more prevalent among patients with ADHD aged ≥ 18 years versus those < 18 years, with the exception of celiac disease (Table 2). These data show an increase in the occurrence of autoimmune diseases in patients with ADHD aged ≥ 18 years compared with those < 18 years.

No greater odds in the ADHD cohorts versus the JIA cohorts were observed across pre-specified autoimmune diseases.

## Discussion

This large, retrospective, cross-sectional study of data from two US healthcare claims databases evaluated 29,215 patients with JIA and 134,625 matched control patients with ADHD aged < 18 years and ≥ 18 years. As expected, the findings suggest that autoimmune diseases are more prevalent in patients of all ages with JIA when compared to patients with ADHD. The results observed from both healthcare claims databases were largely similar.

Patients with one autoimmune disease may be at a higher risk of developing a second one [43]. While a few studies have quantified the occurrence of co-existing autoimmune diseases in patients with JIA, to our knowledge, no large studies have been performed [22–28]. In a single-center study in Italy of 79 patients with JIA (aged ≤ 21 years), 15.2% of patients had at least one autoimmune disease in addition to JIA, with autoimmune thyroid diseases, such as Graves’ disease, being the most common (10.1%) [28]. Crohn’s disease was present in 3.8% of patients with JIA in this study [28]. In another similar study in patients with JIA (aged ≤ 17 years), 11.9% of patients had autoimmune thyroiditis and 6.6% of patients had celiac disease [22]. However, in this present study, autoimmune thyroiditis and celiac disease were reported in < 1% of patients, and Crohn’s disease had a prevalence rate of 0.07–1.52%. The differences observed between these studies and ours are likely due to variations in methodological approach: previous studies screened for co-existing autoimmune diseases by measuring thyroid functionality and autoantibodies in blood samples. In patients with positive autoantibody profiles, diagnosis of Crohn’s disease or celiac disease was confirmed by a small bowel biopsy. Hence, these specific testing methods increase the likelihood of diagnosis of a co-existing autoimmune disease compared with reported ICD codes. However, consistently with the ≥ 18-year-old cohort data reported in our study, a database study in Taiwan (patients with JIA, *n* = 262; participants without JIA, *n* = 107,171), quantifying the development of adult-onset rheumatic diseases among patients with JIA aged ≥16 years, noted increased hazard ratios for ankylosing spondylitis, psoriasis, Sjögren’s syndrome, SLE, and psoriatic arthritis [44].

In a prospective observational study investigating comorbidities in 344 adult patients enrolled in the Juvenile arthritis Methotrexate Biologics long-term Observation (JuMBO) registry, the following autoimmune conditions were reported to co-occur in patients with JIA: uveitis (17.7%), psoriasis (6.4%), Hashimoto’s thyroiditis (1.2%), Crohn’s disease (1.2%), ulcerative colitis (0.9%), and diabetes mellitus (0.9%) [32]. While the rates of gastrointestinal autoimmune diseases reported in JuMBO were comparable with those in our study, the rates of all other autoimmune diseases reported were higher in JuMBO. As noted above, the differences observed may be due to the way in which conditions were identified; patients from the JuMBO register were asked to self-report comorbid conditions and it has been noted that individuals may have different severity thresholds for reporting conditions. It is highly possible that comorbidity was overestimated by Raab et al. [32] as only severely affected patients were included, meaning the data were not representative of the general JIA population.

Although higher odds for the majority of autoimmune diseases were seen in the < 18 years cohort compared with the ≥ 18 years cohort (more patients with JIA than ADHD had a co-existing autoimmune disease), it must be noted that the prevalence rates in the older cohort were generally greater than those in the < 18 years cohort (more patients with both JIA or ADHD had a co-existing autoimmune disease). This may reflect that the older patients had a greater amount of time to develop autoimmune diseases compared with the younger cohort prior to enrollment in this study. The large ORs observed in the patients aged < 18 years can be attributed to the lower prevalence of autoimmune diseases among patients with ADHD compared with patients with JIA. These ORs indicate that patients with JIA are predisposed to the earlier development of these conditions.

Among patients with JIA, 23–54% also had a diagnosis of RA. This is expected as, despite the fact the diseases are two distinct entities, non-pediatric rheumatologist physicians may give a diagnosis of RA, in particular when a patient with JIA moves from pediatric care to an adult healthcare setting [41, 42]. A large, cross-sectional study investigating the prevalence of 37 autoimmune diseases in patients with RA or osteoarthritis revealed that patients with RA had a greater number of concurrent autoimmune diseases [15]. The most prevalent co-existing autoimmune conditions in the patients with RA were SLE and psoriatic arthritis. Diagnosis codes for both SLE and psoriatic arthritis were commonly reported in the JIA population of the present study, reflecting the similarities in the pathogenesis of JIA and RA. In the control osteoarthritis population, as in the ADHD population of our study, type 1 diabetes mellitus was the most highly prevalent co-existing autoimmune disease [15].

The co-existence of multiple autoimmune diseases in patients with JIA may be explained by the involvement of common genes. Autoimmune diseases are significantly more common among relatives of patients with JIA than among healthy controls [45], and a number of associations between human leukocyte antigen alleles and JIA, RA, celiac disease, and type 1 diabetes mellitus have been reported [46]. In addition, a study investigating the alleles and genotypes of patients with JIA and healthy controls revealed strong associations between JIA and variants in certain regions of *TNFAIP3, STAT4* and C*12ORF30* [47]. These regions have previously been associated with a number of other autoimmune diseases including RA and SLE [47].

The limitations of this study should be considered. Firstly, the ICD codes used in administrative data do not allow for distinction between the subcategories of JIA, which may have some influence on the prevalence of co-existing autoimmune diseases reported using these data. Although patients with JIA and ADHD may have a similar number of healthcare encounters, their primary providers are likely to be different (e.g. rheumatologist versus psychiatrist), which may introduce bias due to misclassification. Further misclassification bias may also have arisen due to diagnostic error caused by the complexity of polyautoimmunity, drug-induced symptom occurrence, or misdiagnosis. In addition, identification of medical events or baseline comorbidities here were restricted to computerized population-based data captured as part of the medical claims; these data can be challenging to verify. A study of hospitalized pediatric patients with rheumatic diseases in Kenya demonstrated 77% agreement in coding between the treating physician and medical records clerk [48]. Similarly, a study in Germany demonstrated that two coders have ICD-10-code agreement in around 2/3 cases [49]. It is also important to consider that autoimmune diseases are complex, diagnoses may change (e.g. arthritis associated with inflammatory bowel disease may initially be diagnosed as JIA if the arthritis presents before gastrointestinal symptoms) and symptoms often have multiple diagnosis codes. Finally, there is the possibility that some patients were included in both databases.

The large number of patients included in these analyses allowed for potential differences in the rates of co-existing autoimmune diseases between patients with JIA and patients with ADHD to be detected effectively. The use of two diagnosis codes reduces the risk that codes were entered in error or that a diagnosis was not confirmed. By including a range of possible diagnostic codes for each autoimmune disease, the likelihood of identifying the conditions from the claims data was maximized. The highly similar results observed between both healthcare claims databases also suggests a good degree of reliability.

## Conclusions

We observed that patients with JIA had a greater prevalence of co-existing autoimmune diseases compared to patients with ADHD. In addition, the large ORs suggest that patients with JIA may be predisposed to the earlier development of autoimmune diseases than the general pediatric population. Management and treatment options for patients with JIA should be considered in the context of any other autoimmune diseases present. The design and analyses of future studies assessing the impact of treatment in patients with JIA need to incorporate the risk of other autoimmune diseases in this population.

## Supplementary information

**Additional file 1: Table S1.** ICD-9-CM and ICD-10-CM codes for pre-specified autoimmune diseases.

**Additional file 2: Table S2.** Baseline characteristics before matching for JIA and ADHD groups.

## Data Availability

All data generated or analyzed during this study are included in this published article/as supplementary information files. Bristol Myers Squibb policy on data sharing may be found at https://www.bms.com/researchers-and-partners/independent-research/data-sharing-request-process.html.

## Abbreviations

ADHD: attention deficit hyperactivity disorder
DMARD: disease-modifying antirheumatic drug
ICD-9-CM: International Classification of Diseases, Ninth Revision, Clinical Modification
ICD-10-CM: International Classification of Diseases, Tenth Revision, Clinical Modification
JIA: juvenile idiopathic arthritis
JuMBO: Juvenile arthritis Methotrexate Biologics long-term Observation
MarketScan: Truven Health MarketScan® Commercial Database
OR: odds ratio
PharMetrics: IMS PharMetrics database
RA: rheumatoid arthritis
SLE: systemic lupus erythematosus
US: United States
