# Supplementary material for: Prevalence of co-existing autoimmune disease in juvenile idiopathic arthritis: a cross-sectional study
Source: Pediatr Rheumatol Online J. 2020 Jun 5;18:43. doi: 10.1186/s12969-020-00426-9 (PMC7275412; doi:10.1186/s12969-020-00426-9)
Supplement: Supplementary file 1 — Additional file 1: Table S1. ICD-9-CM and ICD-10-CM codes for pre-specified autoimmune diseases. [file 12969_2020_426_MOESM1_ESM.docx]

**Supplementary Table 1** ICD-9-CM and ICD-10-CM codes for pre-specified autoimmune diseases

| **Autoimmune disease** | **ICD-9-CM code** | **ICD-10-CM code** |
| --- | --- | --- |
| Addison’s disease | 255.4x | E27.1,E27.2,E27.40,E27.49 |
| Alopecia areata | 704.01 | L63.xx |
| Celiac disease | 579.0 | K90.0 |
| Chronic glomerulonephritis | 582.xx | N03.2,N03.3,N03.5,N03.8,N03.9,N08 |
| Chronic urticaria | 708.xx | L50.xx |
| Crohn’s disease | 555.xx | K50.xx |
| Erythema nodosum | 695.2 | L52 |
| Giant cell arteritis | 446.5 | M31.6,M31.5 |
| Graves’ disease | 242.0x | E05.0x |
| Hashimoto’s thyroiditis/autoimmune thyroid disease | 245.2 | E06.3 |
| Hemolytic anemia | 283.xx,282.xx | D59.xx, D58.xx, D57.xx, D56.xx, D55.xx |
| Interstitial lung disease/pulmonary fibrosis | 515.xx, 516.34, 516.69,516.9,516.31 | J84.89, J84.84xx,J84.9,J84.1x |
| Morphea | 701.0 | L94.0 |
| Multiple sclerosis | 340 | G35 |
| Myasthenia gravis | 358.00,358.01 | G70.00,G70.01 |
| Pernicious anemia | 281.0 | D51.0 |
| Polymyalgia rheumatica | 725 | M35.3 |
| Polymyositis | 710.4 | M33.2x |
| Primary biliary cirrhosis | 571.6 | K74.3 |
| Psoriasis only | 696.1,696.8 | L40.1,L40.2,L40.3,L40.4,L40.8,L40.9,L44.8 |
| Raynaud’s syndrome | 443.0 | I73.0x |
| Scleritis/episcleritis | 379.0x | H15.0x,H15.1x |
| Sjögren’s/Sicca syndrome | 710.2 | M35.0x |
| Systemic sclerosis/scleroderma | 710.1 | M34.xx |
| Thrombocytopenic purpura/immune thrombocytopenic purpura | 287.31,287.30 | D69.3,D69.4xx,,D69.5xx,D69.6 |
| Type 1 diabetes mellitus | 250.x1,250.x3 | E10.xx |
| Ulcerative colitis | 556.xx | K51.xx |
| Uveitis | 364.xx | H20.00,H20.019,H20.029,H20.039,H20.049,H20.059,H20.13,H20.9,H20.819,H40.40X0,H20.23,H20.829,H20.9,H21.03,H21.1X9,  H21.269,H21.259,H21.239,H21.249,  H21.279,H21.219,H21.229,H21.29,H21.309,H21.329,H21.319,H21.349,H21.359,  H21.509,H21.549,H21.519,H21.529,H21.43,H21.569,H21.539,H21.559,H21.81,H21.82,H21.89,H21.9 |
| Vasculitis | 709.1,695.81,447.6 | L95.xx, I77.6 |
| Vitiligo | 709.01, 624.8,374.53 | L80, H02.73,N90.89 |

*ICD-9-CM* International Classification of Diseases, Ninth Revision, Clinical Modification, *ICD-10-CM* International Classification of Diseases, Tenth Revision, Clinical Modification.
