# Supplementary material for: Prevalence of co-existing autoimmune disease in juvenile idiopathic arthritis: a cross-sectional study
Source: Pediatr Rheumatol Online J. 2020 Jun 5;18:43. doi: 10.1186/s12969-020-00426-9 (PMC7275412; doi:10.1186/s12969-020-00426-9)
Supplement: Supplementary file 2 — Additional file 2: Table S2. Baseline characteristics before matching for JIA and ADHD groups. [file 12969_2020_426_MOESM2_ESM.docx]

**Supplementary Table 2** Baseline characteristics before matching for JIA and ADHD groups

| **Characteristic** | **< 18 years** | | | | **≥ 18 years** | | | |
| --- | --- | --- | --- | --- | --- | --- | --- | --- |
|  | **MarketScan** | | **PharMetrics** | | **MarketScan** | | **PharMetrics** | |
|  | **JIA**  **(N = 8633)** | **ADHD**  **(N = 616,904)** | **JIA**  **(N = 9155)** | **ADHD**  **(N = 694,965)** | **JIA**  **(N = 5790)** | **ADHD**  **(N = 539,162)** | **JIA**  **(N = 5638)** | **ADHD**  **(N = 611,800)** |
| **Female** | 6173 (71.5) | 184,805 (30.0) | 6587 (71.9) | 208,736 (30.0) | 4507 (77.8) | 281,145 (52.1) | 4315 (76.5) | 316,656 (51.8) |
| **Age, mean (SD)** | 10.6 (4.5) | 11.3 (3.5) | 10.5 (4.5) | 11.1 (3.5) | 37.0 (16.6) | 33.7 (13.1) | 34.6 (14.7) | 33.4 (12.6) |
| **Medications** |  |  |  |  |  |  |  |  |
| bDMARD | 1287 (14.9) | 134 (0.0) | 1485 (16.2) | 154 (0.0) | 1593 (27.5) | 2160 (0.4) | 1614 (28.6) | 2281 (0.4) |
| Non-bDMARD | 2512 (29.1) | 6782 (1.1) | 3127 (34.2) | 7157 (1.0) | 2143 (37.0) | 14,544 (2.7) | 2266 (40.2) | 16,401 (2.7) |
| Corticosteroids* | 2984 (34.6) | 111,296 (18.0) | 3367 (36.8) | 140,184 (20.2) | 2681 (46.3) | 141,869 (26.3) | 2712 (48.1) | 167,219 (27.3) |
| NSAIDs | 4090 (47.4) | 39,500 (6.4) | 4812 (52.6) | 58,067 (8.4) | 2528 (43.7) | 130,908 (24.3) | 2634 (46.7) | 155,733 (25.5) |
| **Comorbidities** |  |  |  |  |  |  |  |  |
| Multiple sclerosis | 1 (0.0) | 89 (0.0) | 1 (0.0) | 86 (0.0) | 20 (0.3) | 1203 (0.2) | 19 (0.3) | 1214 (0.2) |
| Asthma | 777 (9.0) | 59,189 (9.6) | 887 (9.7) | 76,765 (11.0) | 472 (8.2) | 37,779 (7.0) | 486 (8.6) | 46,223 (7.6) |
| Anxiety | 295 (3.4) | 74,822 (12.1) | 317 (3.5) | 94,601 (13.6) | 555 (9.6) | 142,679 (26.5) | 549 (9.7) | 182,936 (29.9) |
| Depression | 208 (2.4) | 52,205 (8.5) | 214 (2.3) | 59,475 (8.6) | 653 (11.3) | 149,377 (27.7) | 622 (11.0) | 182,023 (29.8) |
| Diabetes mellitus | 92 (1.1) | 2851 (0.5) | 84 (0.9) | 3137 (0.5) | 440 (7.6) | 20,442 (3.8) | 371 (6.6) | 21,116 (3.5) |
| Anemia | 498 (5.8) | 6321 (1.0) | 479 (5.2) | 7969 (1.1) | 948 (16.4) | 24,087 (4.5) | 655 (11.6) | 26,888 (4.4) |
| Hypertension | 2 (0.0) | 136 (0.0) | 6 (0.1) | 225 (0.0) | 103 (1.8) | 6993 (1.3) | 103 (1.8) | 8729 (1.4) |
| Hyperlipidemia | 174 (2.0) | 12,885 (2.1) | 202 (2.2) | 18,842 (2.7) | 1162 (20.1) | 91,457 (17.0) | 1023 (18.1) | 107,150 (17.5) |
| Malignancy | 56 (0.6) | 1458 (0.2) | 49 (0.5) | 1413 (0.2) | 278 (4.8) | 10,707 (2.0) | 162 (2.9) | 11,096 (1.8) |
| **CCI score, mean (SD) (49)** | 2.4 (0.6) | 2.1 (0.4) | 2.4 (0.6) | 2.1 (0.4) | 2.9 (1.0) | 2.2 (0.6) | 2.8 (0.9) | 2.2 (0.6) |
| **Autoimmune diseases**^†^ |  |  |  |  |  |  |  |  |
| 0 | 4988 (57.8) | 601,214 (97.5) | 5194 (56.7) | 676,302 (97.3) | 1974 (34.1) | 503,248 (93.3) | 2029 (36.0) | 571,366 (93.4) |
| 1 | 2830 (32.8) | 15,035 (2.4) | 3075 (33.6) | 17,954 (2.6) | 2749 (47.5) | 31,558 (5.9) | 2641 (46.8) | 35,623 (5.8) |
| 2 | 659 (7.6) | 598 (0.1) | 745 (8.1) | 648 (0.1) | 796 (13.8) | 3557 (0.7) | 722 (12.8) | 3949 (0.7) |
| 3 | 127 (1.5) | 51 (0.0) | 121 (1.3) | 54 (0.0) | 190 (3.3) | 624 (0.1) | 190 (3.4) | 702 (0.1) |
| >3 | 29 (0.3) | 6 (0.0) | 20 (0.2) | 7 (0.0) | 81 (1.4) | 175 (0.0) | 56 (1.0) | 160 (0.0) |
| **Healthcare encounters**^‡^ |  |  |  |  |  |  |  |  |
| 0–10 | 4927 (57.1) | 473,994 (76.8) | 3205 (35.0) | 455,054 (65.5) | 2714 (46.9) | 367,139 (68.1) | 1656 (29.4) | 313,550 (51.3) |
| 11–20 | 2353 (27.3) | 84,118 (13.6) | 3352 (36.6) | 143,717 (20.7) | 1567 (27.1) | 96,359 (17.9) | 1741 (30.9) | 156,390 (25.6) |
| 21–30 | 739 (8.6) | 29,751 (4.8) | 1393 (15.2) | 48,196 (6.9) | 730 (12.6) | 38,357 (7.1) | 1008 (17.9) | 67,573 (11.0) |
| 31–40 | 294 (3.4) | 13,731 (2.2) | 604 (6.6) | 21,289 (3.1) | 371 (6.4) | 18,308 (3.4) | 561 (10.0) | 33,442 (5.5) |
| 41–50 | 153 (1.8) | 7130 (1.2) | 262 (2.9) | 11,224 (1.6) | 183 (3.2) | 9373 (1.7) | 273 (4.8) | 18,150 (3.0) |
| > 50 | 167 (1.9) | 8180 (1.3) | 339 (3.7) | 15,485 (2.2) | 225 (3.9) | 9626 (1.8) | 399 (7.1) | 22,695 (3.7) |

Data are shown as n (%) unless otherwise specified.

*By any route of administration.

^†^Includes all autoimmune diseases outlined in Supplementary Table 1, rheumatoid arthritis, ankylosing spondylitis, psoriatic arthritis, dermatomyositis, systemic lupus erythematosus, and sarcoidosis.

^‡^In 1 year.

*ADHD* attention deficit hyperactivity disorder, *bDMARD* biologic disease-modifying antirheumatic drug, *CCI* Charlson Comorbidity Index (49), *JIA* juvenile idiopathic arthritis, *MarketScan* Truven Health MarketScan® Commercial Database, *NSAID* nonsteroidal anti-inflammatory drug, *PharMetrics* IMS PharMetrics database, *SD* standard deviation.
